# Isochlorogenic Acid C Alleviates Allergic Asthma via Interactions Between Its Bioactive Form and the Gut Microbiome

**DOI:** 10.3390/ijms26104864

**Published:** 2025-05-19

**Authors:** Jing-Yi Xu, Xiao-Juan Rong, Zhen Shen, Yun-Dan Guo, Yi-Xuan Zhang, Chen-Chen Ding, Yi Wang, Yan-Xing Han, Tian-Le Gao, Cai Tie

**Affiliations:** 1 State Key Laboratory for Fine Exploration and Intelligent Development of Coal Resources & School of Chemical and Environmental Engineering, China University of Mining and Technology-Beijing, Ding 11 Xueyuan Road, Beijing 100083, China; 18873361988@163.com (J.-Y.X.); 17788266130@163.com (Y.-X.Z.); 13574319616@163.com (C.-C.D.); sycamorey11@163.com (Y.W.); 2 Xinjiang Institute of Material Medica, Urumqi 830004, China; rxj1125@stu.xjmu.edu.cn; 3 State Key Laboratory of Bioactive Substances and Function of Natural Medicine, Institute of Materia Medica, Chinese Academy of Medical Sciences & Peking Union Medical College, Beijing 100050, China; shenzhen@imm.ac.cn (Z.S.); guoyundan@imm.ac.cn (Y.-D.G.); hanyanxing@imm.ac.cn (Y.-X.H.)

**Keywords:** isochlorogenic acid C, asthma, airway inflammation, docosahexaenoic acid, lipid metabolomics

## Abstract

The global prevalence of asthma is approximately 4.3%, and current asthma treatments focus on reducing symptoms, maintaining normal activity levels, and preventing the deterioration of lung function, rather than achieving a cure or complete prevention. We identified isochlorogenic acid C (ICGAC) as a potential natural medicine for the treatment of asthma. However, the bioavailability of ICGAC was low, ranging from 14.4% to 16.9%, suggesting the involvement of the gut microbiota. The full spectrum of ICGAC’s anti-asthmatic mechanism remains to be elucidated. This study investigated the mechanism by which ICGAC alleviates allergic asthma through the gut–lung axis. We discovered anti-asthma pathways and targets based on the selective regulation of lipid peroxidation and employed pharmacological tools to preliminarily validate their mechanisms and efficacy. To study the role of ICGAC in regulating the gut microbiota, we performed 16S rRNA gene sequencing and metabolite analysis. Furthermore, by combining molecular biology and lipid metabolomics, we elucidated the underlying anti-asthma mechanisms of ICGAC. The effective form of ICGAC varies between single and long-term administration. The oral administration of ICGAC enhances the gut-microbiota-derived production of short-chain fatty acids (SCFAs) as the active substances, modulates immune cell activity, influences the differentiation of T- and B-cells, and reduces airway inflammation. ICGAC also regulates the metabolic network of lipid mediators (LMs) and polyunsaturated fatty acids (PUFAs), thus exerting anti-inflammatory effects by modulating arachidonate lipoxygenase (ALOX) activity and LM levels. In addition, ICGAC enhanced the antioxidant response by upregulating the expression of glutathione peroxidase 4 (GPX4), solute carrier family 7 member 11 (SLC7A11), and nuclear factor erythroid 2-related factor 2 (Nrf2), while inhibiting the release of interleukin-4 (IL-4), thereby suppressing asthma inflammation and IgE production. The anti-asthmatic mechanism of oral ICGAC involves the inhibition of lipid peroxidation by chlorogenic acid (CGA) and SCFAs produced by the gut microbiota. ICGAC suppresses asthma-associated inflammatory and oxidative stress responses through the upregulation of GPX4, SLC7A11, and Nrf2 in lung tissue. This study not only provides a solid foundation for the potential clinical use of ICGAC in asthma treatment but also offers novel insights for future research and therapeutic strategies targeting asthma.

## 1. Introduction

According to the latest data from the World Health Organization in 2019, asthma has a global incidence rate of 3%, affecting over 600 million individuals with symptoms, resulting in 461,000 deaths annually [1]. In China, the incidence rate stands at 4.2% [2]; it is characterized by a large patient base, high incidence, and high deterioration rates. However, challenges such as insufficient diagnosis and treatment persist, leading to reduced quality of life and posing a significant financial burden, particularly for those with poorly controlled asthma and in low-income settings [3].

Marked by airway inflammation, hyperresponsiveness, and airway remodeling, asthma is a condition closely associated with exposure to allergens or diverse physical and chemical stimuli. Its clinical presentation includes wheezing, coughing, shortness of breath, chest tightness, and breathing difficulties, significantly disrupting patients’ daily activities and overall quality of life [4]. In more severe cases, asthma can damage airways and impair oxygen flow to the alveoli, potentially leading to life-threatening complications [3]. Influenced by both genetic and environmental factors, including infections, allergens, and irritants, asthma exhibits heterogeneity in its inflammatory and remodeling processes [5]. Given its complexity, asthma treatment aims to alleviate symptoms, preserve normal activity levels, and prevent the deterioration of lung function, rather than achieving a complete cure or absolute prevention [6].

Asthma therapy is guided by a stepwise approach tailored to disease severity and control, as recommended by global guidelines [7]. All patients require immediate access to a rapid-onset bronchodilator (e.g., short-acting β_2_-agonists [SABAs]) for symptom relief. For mild intermittent asthma, as-needed SABA use may suffice, while patients with persistent symptoms or exacerbations require controller medications. First-line controllers include low-dose inhaled glucocorticoids (IGCs), which reduce airway inflammation and are the cornerstone of long-term management. For moderate disease, IGCs are often combined with long-acting β_2_-agonists (LABAs) in a single inhaler (fixed-dose combination) to improve adherence and efficacy [8]. Alternative options for mild-to-moderate asthma include leukotriene receptor antagonists (LTRAs) or theophylline.

Severe asthma warrants high-dose IGCs/LABA combinations, and patients should be evaluated for add-on therapies such as long-acting muscarinic antagonists (LAMAs) or biologic agents. Systemic glucocorticoids should be avoided whenever possible; instead, patients with eosinophilic or type 2 inflammation (e.g., elevated blood eosinophils or FeNO) may benefit from targeted biologics such as anti-IgE (omalizumab), anti-IL-5/IL-5R (mepolizumab, reslizumab, benralizumab), or anti-IL-4/IL-13 (dupilumab) [9]. These therapies address specific inflammatory pathways and reduce the risk of exacerbation, aligning with the principles of personalized medicine [10].

However, these treatments have limitations, such as the requirement for lifelong use, ineffectiveness in some patients, and potential side effects including suppression of the hypothalamic–pituitary–adrenal axis, growth retardation, osteoporosis, diabetes, and respiratory infections [11]. Therefore, there is a significant unmet clinical demand for asthma therapies that are both highly effective and safe. This has prompted a shift towards exploring natural medicines, which offer a plethora of potential drug candidates with minimal adverse effects. These natural compounds often exhibit multiple therapeutic advantages, despite their complex mechanisms of action and potentially unidentified primary targets [12,13].

In pursuit of more effective and safer asthma therapies, our research has focused on *Hyssopus cuspidatus* Boriss, a perennial herb of the Labiatae family with traditional uses in treating colds, fever, phlegm, and cough. Its anti-allergic inflammation properties have been reported in mouse asthma and anaphylaxis models, with Isochlorogenic acid C (ICGAC) identified as a primary bioactive component [14,15]. This dicaffeoylquinic acid, belonging to the caffeoylquinic class of acids, exhibits a wide range of pharmacological effects such as antioxidant, anti-inflammatory, and antimicrobial activities [14]. Preclinical studies have shown that ICGAC can inhibit eosinophil activation and migration, suppress histamine release from mast cells, and restore the Th1/Th2 immune balance, thereby effectively alleviating asthma symptoms [16]. Although the therapeutic potential of ICGAC in asthma is evident, its precise mechanisms of action remain to be fully elucidated, particularly given its low bioavailability, which ranges from 14.4% to 16.9%. This suggests a potential role for the gut microbiota in mediating some of these effects, given its critical involvement in immune and inflammatory regulation, as well as its emerging implications in respiratory diseases [14].

Lipidomics, which involves the comprehensive analysis of thousands of lipid species and their functions, has revealed that lipids, as crucial signaling molecules, modulate various cellular processes in asthma patients’ airways, influencing disease onset and progression [17]. Notably, asthma has been associated with alterations in glycerophospholipid and sphingomyelin metabolism, leading to the discovery of potential lipid biomarkers such as PC(18:1/18:2), PC(16:0/18:1), and PC(18:0/22:5), serving as valuable references in the research and development of asthma treatments [18].

The aim of this study is to elucidate the anti-asthma mechanism of ICGAC by adopting an integrative gut–lung axis approach. Employing an Ovalbumin (OVA)-induced asthma mouse model, we place special emphasis on deciphering how ICGAC interacts with the gastrointestinal system when exerting its anti-asthmatic effects, particularly focusing on the gut microbiota and intestinal epithelial cells. Through the application of lipid metabolomics and network pharmacology, we aim to uncover the multifaceted mechanisms of ICGAC in asthma, including its regulation of the lipid metabolism, modulation of inflammatory mediators, immune cell function, and airway responsiveness. Ultimately, this research aims to lay a scientific foundation for the potential clinical application of ICGAC in asthma management.

## 2. Result

### 2.1. Histopathological Analysis of Lungs

**Figure 1:**
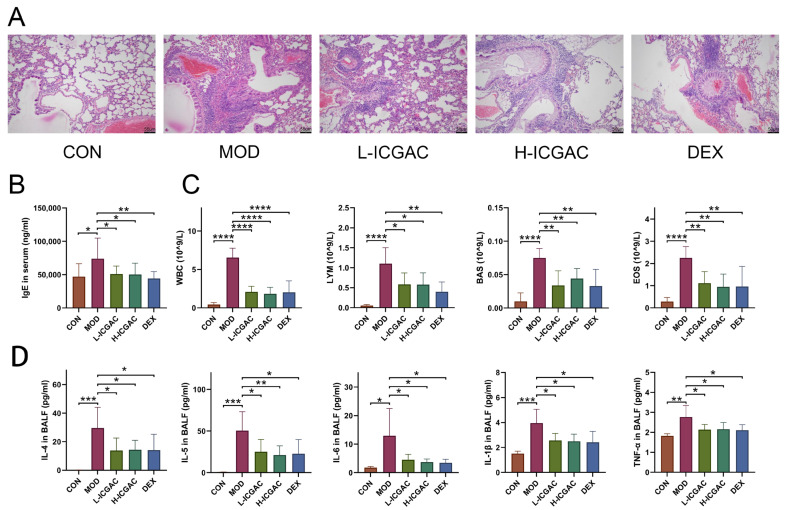
(**A**) Lung histology analysis of different groups. (**B**) IgE levels in the serum of mice. (**C**) The number of white blood cells (WBC), lymphocytes (LYM), basophils (BAS), and eosinophil (EOS) in the BALF. (**D**) IL-4, IL-5, IL-6, IL-1beta, and TNF-alpha levels in the supernatant of BALF. Data are shown as the mean ± SEM (n = 5–8), * *p* < 0.05, ** *p* < 0.01, *** *p* < 0.001, **** *p* < 0.0001.

We assessed lung tissue morphology using H&E staining and light microscopy to compare the effects of ICGAC and dexamethasone (DEX) on asthma-induced structural alterations in mice. As shown in Figure 1A, the control group displayed normal lung tissue architecture with no notable inflammatory infiltration. In contrast, the model group exhibited marked inflammatory cell infiltration around the bronchi and blood vessels, along with bronchial epithelial degeneration, bronchial obstruction, alveolar wall thickening, and interstitial inflammatory cell infiltration. The peribronchial and perivascular inflammatory infiltrates in OVA-induced asthma mice consisted of eosinophils and mast cells, along with lymphocytic involvement [19]. Treatment with both DEX and ICGAC significantly attenuated these pathological changes, including inflammatory cell infiltration and alveolar wall thickening, highlighting their protective roles in mitigating asthma-induced lung damage. The analysis of the pathological results of lung injury in mice is shown in Table S1. In the L-ICGAC and H-ICGAC groups, the mice still had inflammatory cell infiltration in the bronchi of the lungs. However, in general, lung injury was reversed to a certain extent after ICGAC administration. The treatment with DEX could also reverse lung tissue injury in asthmatic mice. In the asthma model group, one mouse (12.5%) had moderate pathological changes in most of the lung tissue, six mice (75%) had moderate pathological changes in part of the lung tissue, and one mouse (12.5%) had mild pathological changes in a small part of the lung tissue. After ICGAC and DEX treatment, no mice with severe pathological changes in the lung tissue were found in the administration groups. After ICGAC treatment, the lung injury in asthmatic mice was reversed and showed a dose-dependent trend. In the low-dose group, seven mice (87.5%) had moderate pathological changes in part of the lung tissue, and one mouse (12.5%) had mild pathological changes in a small part of the lung tissue. In the high-dose group, only two mice (25%) had moderate pathological changes in part of the lung tissue, and six mice (75%) had mild pathological changes in a small part of the lung tissue.

### 2.2. Modulatory Effects of ICGAC on Immune Cells and Biochemical Cytokines

As shown in Figure 1B, serum IgE levels in the model group were significantly elevated compared to the control group, confirming the successful induction of the mouse asthma model through OVA sensitization. Treatment with DEX and ICGAC effectively reversed the abnormal increase in IgE levels. Notably, the IgE concentrations in the L-ICGAC (low-dose ICGAC) and H-ICGAC (high-dose ICGAC) groups were comparable, indicating no significant dose-dependent effect of ICGAC on IgE reduction.

The numbers of various immune cells (WBC, LYM, EOS, MON) are shown in Figure 1C. In the model group, the number of immune cells was increased relative to the control group. After treatment with both DEX and ICGAC acid, there was a significant reduction in the number of immune cells. Moreover, we did not observe significant differences between the L-ICGAC and H-ICGAC groups in the assessed indicators. From each group of mice, bronchoalveolar lavage fluid (BALF) was collected. Asthma-associated type 2 cytokines (IL-4, IL-5, IL-6, IL-1β and TNF-α) in BALF are shown in Figure 1D. All cytokines were higher in the model group than in the control group. Inflammatory cytokines (including IL-4, IL-5, IL-6, IL-1β and TNF-α) were substantially reversed after treatment with the ICGAC and DEX. However, there was no significant difference in cytokine levels between the L-ICGAC and H-ICGAC groups, suggesting a lack of dose-dependent effects in cytokine modulation.

### 2.3. Differences in Effective Substance Profiles in Serum Between Long-Term and Single-Dose ICGAC Treatments for Asthma in Mice

**Figure 2:**
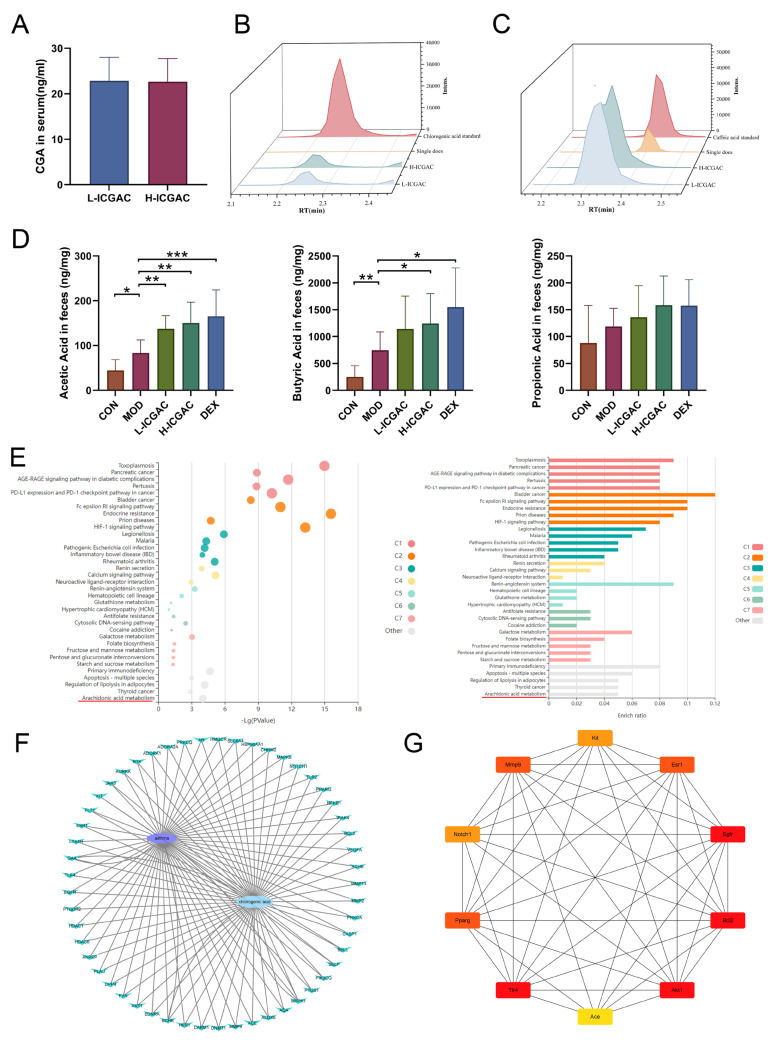
(**A**) CGA quantity in serum. (**B**) Chromatogram of chlorogenic acid (L−ICGAC, H−ICGAC, single does, and chlorogenic acid standard). (**C**) Chromatogram of caffeic acid (L−ICGAC, H−ICGAC, single does, and caffeic acid standard). (**D**) Quantitative results of acetate acid, propionate acid, and butyrate acid concentrations in feces. (**E**) KEGG analysis for the signaling pathways and metabolic pathways of potential target genes. (**F**) Network of CGA and asthma compound (ingredient) target network (**G**) Network of CGA and asthma TOP10 compound (ingredient) target network. Data are shown as the mean ± SEM (n = 6–8), * *p* < 0.05, ** *p* < 0.01, *** *p* < 0.001.

We quantified the concentrations of ICGAC, chlorogenic acid, and caffeic acid in serum samples from long-term-treated mice using UPLC-MS. ICGAC was not detected in the control group. However, chlorogenic acid was identified in the serum of both the L-ICGAC and H-ICGAC groups. As illustrated in Figure 2A, the levels of chlorogenic acid were comparable between the L-ICGAC and H-ICGAC groups, with concentrations of 22.64 ng/mL and 22.85 ng/mL, respectively. These findings indicate that chlorogenic acid is the effective substance in serum following the long-term administration of ICGAC.

We measured the concentrations of ICGAC, chlorogenic acid, and caffeic acid in serum samples from single-dose-treated mice using UPLC-MS. Neither ICGAC nor chlorogenic acid was detected in the control or single-dose groups. However, caffeic acid was identified in the serum of the single-dose groups (Figure 2B,C). These results suggest that caffeic acid is the primary effective substance in serum following the single-dose administration of ICGAC.

### 2.4. ICGAC and DEX Influence the Concentration of SCFAs Produced by Gut Microbiota in Asthmatic Mice

In our study, we determined SCFA levels in the feces using GC-MS (Figure 2D). The current study showed that the levels of acetic acid, propionic acid, and butyric acid in feces were higher in the model group of mice compared to the control group. Meanwhile, the DEX, H-ICGAC and L-ICGAC groups had even higher levels of acetic acid, propionic acid, and butyric acid than the model group. Specifically, acetic acid and butyric acid were significantly up-regulated following the ICGAC treatment. The highest level of acetic acid was observed in the H-ICGAC group, while propionic acid levels were comparable between the H-ICGAC and DEX groups. The highest concentration of butyric acid was detected in the DEX group. These findings highlight the role of ICGAC and DEX in enhancing SCFA production, potentially contributing to their therapeutic effects in asthma.

### 2.5. Gut Microbiota Metabolizes ICGAC into Chlorogenic Acid, Its Active Form, Modulating Asthma Pathways

Chlorogenic acid was identified as the active metabolite form of ICGAC responsible for controlling allergic asthma, as demonstrated through a network pharmacology analysis. Kyoto Encyclopedia of Genes and Genomes (KEGG) pathway enrichment analysis showed that multiple signaling pathways and metabolic pathways, including asthma-related arachidonic acid metabolism pathways, were significantly affected (Figure 2E). Targets related to asthma were obtained from DisGeNET (https://disgenet.com/; access date: 17 January 2025), GeneCards, Home (https://www.genecards.org/; access date: 17 January 2025), OMIM (https://secure.jhu.edu/form/OMIM; access date: 17 January 2025) and e-TSN (http://www.lilab-ecust.cn/etsn/; access date: 17 January 2025). Targets related to chlorogenic acid were obtained from Swiss Target Prediction (http://www.swisstargetprediction.ch/; access date: 17 January 2025) and BATMAN (http://bionet.ncpsb.org.cn/batman-tcm/#/home; access date: 17 January 2025). The correlation analysis between the targets of chlorogenic acid and targets of asthma identified 51 potential targets to construct a PPI network using Cytoscape v3.8.2 (Figure 2F). The top 10 genes were selected as hub genes, including RAC-alpha serine/threonine-protein kinase 1 (Akt1), Peroxisome Proliferator-Activated Receptor Gamma (Pparg), Epidermal Growth Factor Receptor (Egfr), Matrix Metalloproteinase 9 (Mmp9), Toll-Like Receptor 4 (Tlr4), Estrogen Receptor 1 (Esr1), KIT Proto-Oncogene Receptor Tyrosine Kinase (Kit), Neurogenic Locus Notch Homolog Protein 1 (Notch1), Angiotensin-Converting Enzyme (Ace), and Bruton’s Tyrosine Kinase (Btk) (Figure 2G).

### 2.6. Effect of ICGAC on the Composition of the Gut Microbiota

**Figure 3:**
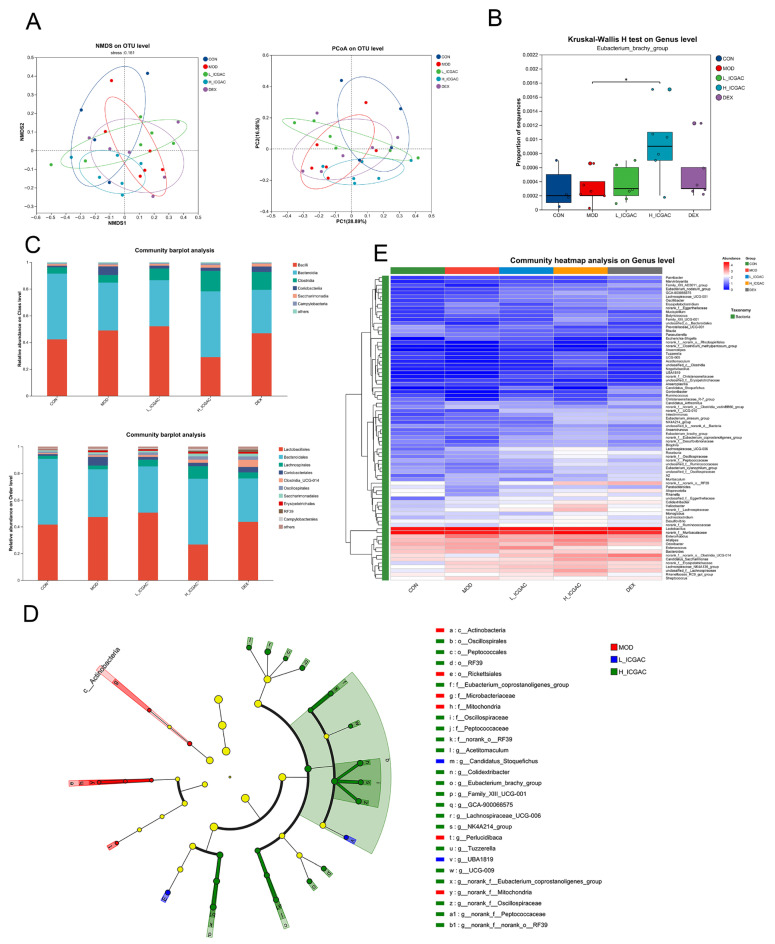
(**A**) Microbiota community analysis based on NMDS and PCoA score plots. (**B**) Quantitative analysis of Eubacterium_brachy_group. (**C**) Community abundance profiling of the gut microbiota on the class (left) and order (right) levels. (**D**) Linear discriminant analysis effect size (LEfSe) analysis for identifying the key enriched bacteria between model group, L−ICGAC group and H−ICGAC group. (**E**) Heatmap analysis of the top 80 differentiated taxa on the genus levels. * *p* < 0.05, when high dose ICGAC group was compared with model group.

In the study, 16S rRNA gene sequencing was performed on fecal samples from each group to investigate how ICGAC modulates the gut microbiota. Principal coordinate analysis (PCoA) and non-metric multidimensional scaling (NMDS) showed that the ICGAC treatment significantly altered the structure of the gut microbiota. However, these changes did not restore the microbiota to a state similar to that of the control group, indicating a distinct directional shift (Figure 3A).

To further confirm which bacterium altered by the ICGAC treatment could in turn affect the disease progression of OVA-induced asthma, we performed high-dimensional categorical comparisons using the linear discriminant analysis (LDA) of effect size (LEfSe), which detected marked differences in the predominance of bacterial communities between the model and ICGAC groups (Figure 3D). The results showed that *Eubacterium_brachy_group* displayed an enrichment in the ICGAC group, which might be associated with the transformation of ICGAC into chlorogenic acid and the subsequent amelioration of asthma symptoms (Figure 3B). Studies have shown that chlorogenic acid has remarkable anti-asthmatic efficacy [20]. Specifically, the abundance of *Clostridia* and *Lachnospirales* increased after the ICGAC treatment (Figure 3C). Overall, treatment with ICGAC induced significant changes in the structure and composition of the gut microbiota in asthmatic mice. Those trends were further supported by a heatmap analysis of the top 80 differentiated taxa on the genus levels (Figure 3E).

### 2.7. ICGAC Regulates Pulmonary Lipid Mediators to Mitigate Asthma Pathogenesis

**Figure 4:**
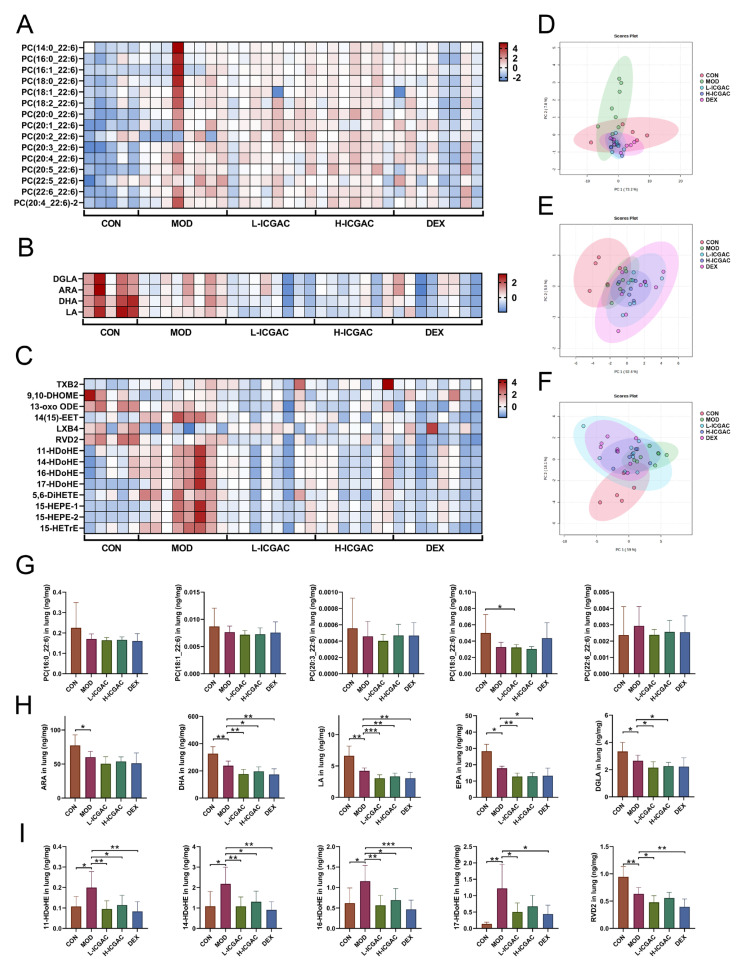
(**A**–**C**) Heatmap of PC (**A**), FFA (**B**) and LMs (**C**) in different groups. (**D**–**F**) PCA of PC (**D**), FFA (**E**) and LMs (**F**) in different groups. (**G**–**I**) PC (X_22:6) quantitation in lungs. (**H**) PUFA (ARA, DHA, LA, EPA, DGLA) quantitation in lungs. (**I**) LMs (11−HDoHE, 14−HDoHE, 16−HDoHE, 17−HDoHE, RVD2) quantitation in lungs. Data are shown as the mean ± SEM (n = 5–8), * *p* < 0.05, ** *p* < 0.01, *** *p* < 0.001.

To confirm the regulation of the phosphatidylcholine (PC) metabolic network by ICGAC, we quantified 206 PCs in the lung using UPLC tandem mass spectrometry. In total, 159 PCs were detected in mouse lung tissue. For the lung tissue, scatter plots based on principal component analysis (PCA) scores showed significant differences in PCs levels between the control and model groups (Figure 4D). PCA showed that there was a significant difference in PCs levels between the control group compared with the model group, with the ICGAC treatment resulting in PCs levels similar to those in the model group. Focusing on PCs containing docosahexaenoic acid (DHA), we observed no significant changes in PC concentrations in the ICGAC group compared to the model group. However, PC concentrations were higher in the DEX group than in the model group (Figure 4G). These trends were further supported by heatmap analysis, as illustrated in Figure 4A.

To confirm the regulation of the polyunsaturated fatty acid (PUFA) metabolic network by ICGAC, we quantified arachidonic acid (ARA), docosahexaenoic acid (DHA), dihomo-γ-linolenic acid (DGLA), eicosapentaenoic acid (EPA), and linoleic acid (LA) in the lung using UPLC tandem mass spectrometry. In lung tissue, scatter plots based on PCA scores showed significant differences in polyunsaturated fatty acids levels between the control and model groups (Figure 4E). Further quantitative results revealed that OVA induction significantly reduced levels of ARA, DHA, DGLA, EPA, and LA. However, the concentration of ARA, DHA, DGLA, EPA, and LA were lower in the ICGAC group than in the model group (Figure 4H). Those trends were further supported by heatmap analysis, as shown in Figure 4B.

We quantified 105 ARA-, DHA-, DGLA-, EPA-, and LA-derived LMs in the lung using UPLC tandem mass spectrometry. In mouse lung tissue, 70 LMs were detected. Scatter plots based on PCA scores demonstrated significant differences in PUFA levels between the control and model groups (Figure 4F). PCA further revealed a significant difference in LM levels between the H-ICGAC and L-ICGAC groups compared to the model group. The ICGAC treatment induced a reversal of asthma-associated alterations in LM metabolism, suggesting a regulatory effect on lipid mediator pathways. Seven LMs (one LM was a metabolite of DGLA, one LM was a metabolite of ARA, one LM was a metabolite of EPA, and five LMs were metabolites of DHA) were determined to be significantly altered in the lungs based on further statistical analysis (Figure 4I). We focused on the DHA metabolites 11-Hydroxydocosahexaenoic acid (11-HDoHE), 14-HDoHE, 16-HDoHE, and resolvin D2 (RVD2), all of which are products of the arachidonate lipoxygenase (ALOX) enzyme. These metabolites were significantly up-regulated following asthmatic modeling. However, after ICGAC treatment, the levels of 11-HDoHE, 14-HDoHE, 16-HDoHE, and RVD2 were significantly down-regulated. These trends were further confirmed by the heatmap analysis, as shown in Figure 4C.

**Figure 5:**
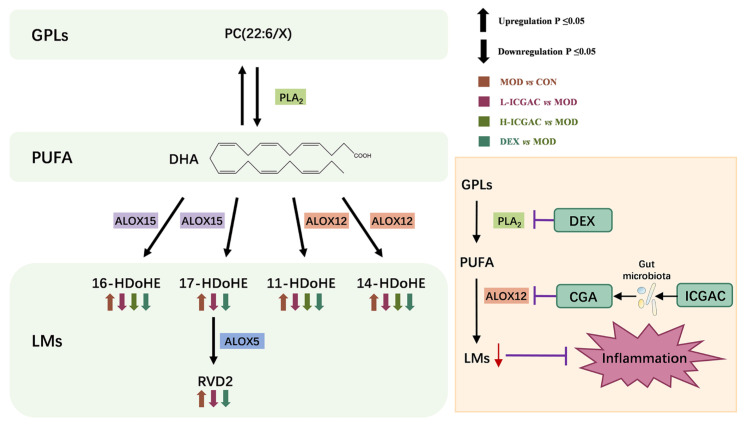
Metabolic pathway of PC and mechanism of anti-asthmatic action of DEX and ICGAC.

As shown in Figure 5, phospholipase A2 (PLA2) catalyzes the hydrolysis of the sn-2 position of PC (22:6/X) to release DHA. ALOX then catalyzes the conversion of DHA into LMs. Dexamethasone reduces LM production by inhibiting PLA2 activity, while ICGAC is metabolized by the gut microbiota to produce chlorogenic acid, which suppresses LM production by inhibiting ALOX12 activity.

### 2.8. ICGAC Modulates ALOX12, SLC7A11, Nrf2, and GPX4 Expression to Enhance Antioxidant Defense in OVA Mice

**Figure 6:**
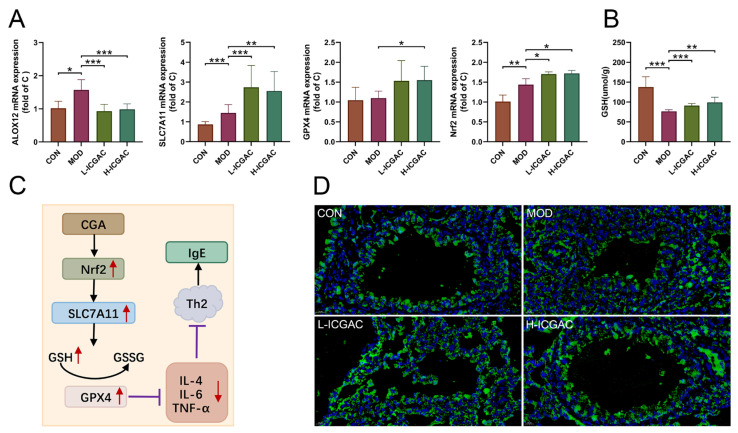
(**A**) The expression of lung ALXO12, SLC7A11, GPX4, Nfr2 using qRT-PCR. (**B**) The level of GSH in lung. Data are shown as the mean ± SEM (n = 3–8), * *p* < 0.05, ** *p* < 0.01, *** *p* < 0.001. (**C**) CGA inhibits the IgE production effect based on the Nrf2/GPx4 pathway. (**D**) Representative photomicrographs obtained using confocal microscopy after the immunofluorescence staining of GPX4. (GPX4 is displayed in green. Nuclei of cells are displayed in blue. scale bar: 100 μm).

We determined ALOX12, solute carrier family 7 member 11 (SLC7A11), nuclear factor erythroid 2-related factor 2 (Nrf2), and GPX4 expression in the lung tissues using real-time quantitative PCR. As shown in Figure 6A, H-ICGAC and L-ICGAC intervention significantly upregulated the expression of SLC7A11, GPX4 and Nrf2. H-ICGAC and L-ICGAC intervention significantly downregulated the expression of ALOX12. GSH levels in the lung tissue of mice were significantly upregulated after ICGAC treatment compared to the model group (Figure 6B). As shown in Figure 6D, ICGAC significantly reversed the OVA-induced downregulation of GPX4. As shown in Figure 6C, ICGAC increased the expression of SLC7A11, promoting the uptake of extracellular cystine into cells for conversion to cysteine. This led to enhanced GSH synthesis, increased GPX4 activity, and the subsequent inhibition of IgE production.

## 3. Material and Method

### 3.1. Material

ELISA kits for the determination of tumor necrosis factor-α (TNF-α), interleukin (IL)-1β, IL-4, IL-5, IL-6, and immunoglobulin E (IgE) were purchased from Cusabio Biotech Co., Ltd. (Wuhan, China). Glutathione (GSH) assay kits were obtained from Nanjing Jiancheng Bioengineering Institute (Nanjing, China). Mass spectrometry (MS)-grade water, acetonitrile, formic acid, acetic acid, ethanol, isopropanol, methyl tert-butyl ether, and methanol were purchased from Thermo Fisher Scientific (Waltham, MA, USA). Glycerol, OVA, and butylated hydroxytoluene (BHT) were purchased from Sigma-Aldrich (St. Louis, MO, USA). Deuterated lipid mediator (LM) standards (IS) were purchased from Cayman (Ann Arbor, MI, USA). Deuterated phosphatidyl choline (PC) standards (IS) were purchased from Avanti (Alabaster, AL, USA). Deuterated propionic acid was purchased from Shanghai Zzbio Co., Ltd. (Shanghai, China). All other reagents were of analytical or high-performance liquid chromatography (HPLC) grade.

### 3.2. Establishment of the Asthma Mouse Model

All animal experiments were conducted in accordance with the guidelines established by the Animal Care and Use Committee (IACUC) of Xinjiang Medical University and the Ethics Committee of the same institution (XJIMM-20230704). After purchase, the animals were kept in an animal house at a constant temperature (22 °C) and 50% relative humidity; they were fed ad libitum and acclimatized for 1 week during the rearing period.

BALB/C female mice (18–22 g) were divided into five groups: a naïve control group (A), a model group (B), a high-dose ICGAC group (C) (2 mg/mL, 20 mg/kg), a low-dose ICGAC group (D) (1 mg/mL, 10 mg/kg), and a dexamethasone (DEX) group (E) (0.16 mg/mL, 1.6 mg/kg). Group A consists of 6 mice, while Groups B, C, D, E, and F each comprise 8 mice. Mice in groups B, C, D, and E were intraperitoneally injected with 0.2 mL of OVA sensitization solution (aluminum hydroxide gel saline dilution 0.5 mg/mL), and mice in group A were injected with an equal amount of saline from days 1–8. On days 15–21, mice in group A were given saline stimulation, and mice in group B, C, D, E were given atomized OVA stimulation solution (OVA 10 mg/mL) for 30 min once a day for 7 days. Groups C, D, E were administered ICGAC and dexamethasone intragastrically. The dose was determined according to the previous study. After 1 h, atomized OVA was used for stimulation, once a day for 30 min per dose for 7 days. After gavage on the first day, three mice were randomly selected as single-dosage groups from the control and high-dose ICGAC groups. During the experiment, the mice were allowed to eat and drink freely.

### 3.3. Collection of Bio-Samples

Serum: Blood serum was collected from mouse eye samples and transferred into Eppendorf tubes. The tubes were then left to sit at room temperature for 2 h, followed by centrifugation at 3000 rpm for 15 min. The serum was subsequently stored at −20 °C for IgE detection and a serum components assay.

Bronchoalveolar lavage fluid (BALF): Mice were euthanized and skinned, followed by immediate isolation of the peritracheal tissues. A transverse incision was made in the upper trachea (between the third and fourth tracheal rings), and a 1 mL syringe needle was inserted into the trachea (approximately 2 mm) and secured. The right main bronchus was ligated, and the left lung was flushed three times with saline, 0.5 mL each time (with a recovery rate of 80–90%). The collected BALF was centrifuged twice at 2000 rpm for 10 min each, and the supernatant was aliquoted into 200 μL portions and stored at −80 °C for a subsequent cytokine assay.

Lung tissues: After serum collection, the mice were dislocated and executed. Lung tissues were immediately removed and rinsed clean with saline. The left lung was placed in a 5 mL Eppendorf tube containing 4% paraformaldehyde solution for morphological analysis. The right lung was immediately frozen in liquid nitrogen for 30 min and then transferred to a −80 °C refrigerator for the later analysis of phosphatidyl cholines (PCs), polyunsaturated fatty acids (PUFAs), and lipid mediators (LMs).

Fecal samples: Fecal samples were collected from the colon and immediately snap-frozen in liquid nitrogen. Subsequently, they were stored at −80 °C for later biochemical analysis, including the determination of short-chain fatty acids (SCFAs).

### 3.4. Biological Sample Preparation for PUFA, LM, and PC Assays

Preparation for the LM Assay: The preparation of lipid mediators begins with homogenizing 5 mg of finely ground lung tissue in 175 μL of acetonitrile through vigorous vortexing for 10 min. After centrifugation at 13,300 rpm for 10 min, the clarified supernatant is carefully collected and combined with stabilizing reagents: 35 μL of 10% glycerol, 1 μL of 10 mg/mL BHT to prevent oxidation, and 10 μL of 50 ng/mL internal standard. The mixture is diluted with 359 μL of 25% acetonitrile and vortexed again to ensure homogeneity. For purification, the sample is passed through a Waters MAX solid phase extraction (SPE) column (Milford, MA, USA) preactivated with acetonitrile and equilibrated with 25% acetonitrile. Sequential washes with 25% acetonitrile and pure acetonitrile eliminate interfering substances, while the target analytes are eluted using 1% formic acid in acetonitrile. The eluate is concentrated under vacuum and stored at −20 °C until analysis. Prior to ultra-high-performance liquid chromatography—multiple reaction monitoring (UHPLC-MRM) detection, the samples are reconstituted in a methanol/acetonitrile solution (50:50, *v*/*v*) to optimize chromatographic compatibility.

Preparation for the PC Assay: Building on the residual material from the LMs assay, phosphatidylcholine extraction involves supplementing the remaining supernatant with 50 μL of methanol and 1 mL of methyl tert-butyl ether (MTBE). The biphasic system is vortexed for 10 min to enhance lipid partitioning, followed by centrifugation at 13,300 rpm to isolate the organic phase. A 200 μL aliquot of the upper layer is transferred and dried under vacuum to remove solvents, with the resulting residue stored at −20 °C for stability. For downstream ultra-high-performance liquid chromatography—high-resolution mass spectrometry (UHPLC-HRMS) analysis, dried PC extracts are resolubilized in acetonitrile containing a 200 ng/mL internal standard.

This sequential methodology not only maximizes sample utilization but also minimizes cross-contamination risks between lipid classes, ensuring analytical specificity for both LMs and PCs within a unified workflow.

### 3.5. Biochemical Assays in Bronchoalveolar Lavage Fluid

The collected BALF was centrifuged at 3500 rpm for 15 min. The cell pellet was suspended in 1 mL phosphate-buffered saline (PBS) for inflammatory cell counting and classification. Levels of basophils (BAS), eosinophils (EOS) white blood cells (WBC), and lymphocytes (LYM) in BALF were measured using an automatic cell counter (Nihon Kohden, Shinjuku, Japan) according to the manufacturer’s protocol. Levels of IL-4, IL-5, IL-6, IL-1β, TNF-α in BALF and the total IgE level in serum were measured using corresponding kits.

### 3.6. Biological Sample Preparation for Active Components Analysis

We mixed 10 µL of serum and 40 µL of methanol containing 0.1% formic acid. After centrifugation at 13,300 rpm for 10 min, 30 µL of the supernatant was taken and then subjected to mass spectrometry analysis.

### 3.7. Biological Sample Preparation for Fecal SCFAs Analysis

In brief, 10 mg of feces was mixed with 600 μL of water containing 0.5% concentrated sulfuric acid. The mixture was vortexed for 5 min and sonicated for 30 min and then centrifuged for 10 min at 13,300 rpm. Then, 500 μL of the supernatant was collected and extracted with 800 μL of MTBE. The mixture was vortexed for 5 min and centrifuged for 10 min at 13,300 rpm. Subsequently, 700 μL of the supernatant was collected and mixed with 0.15 g of anhydrous sodium sulfate. From this, another 500 μL aliquot was taken and combined with 500 μL of Propionic Acid-d3 (200 μM). After vortexing for another 5 min, the mixture was transferred to a chromatographic vial for analysis.

### 3.8. Real-Time Quantitative PCR Analysis

Total RNA was extracted from lung tissues using the RNAeasy™ Animal RNA Isolation Kit with Spin Column (Beyotime, Shanghai, China). The concentration of total RNA was quantified using a NanoDrop 2000 spectrophotometer (Thermo Fisher Scientific, Waltham, MA, USA). Then, 1 μg of purified RNA from each sample was reverse-transcribed into complementary DNA (cDNA) using the HiFi-Script cDNA Synthesis Kit (CWBIO, Taizhou, Jiangsu, China). Quantitative reverse transcription PCR (qRT-PCR) was performed using an ABI 7500 Fast PCR system (Applied Biosystems, San Francisco, CA, USA) using UltraSYBR Mixture (Low ROX) (CWBIO, Taizhou, China). Glyceraldehyde-3-phosphate dehydrogenase (GAPDH) was employed as an internal control for normalization. The relative expression levels were calculated as fold changes in comparison to GAPDH, using the 2^−ΔΔCT^ method.

### 3.9. The Determination of Glutathione (GSH)

First, we accurately weighed the tissue (approximately 0.005 g) and proceeded to homogenize it in physiological saline using a weight-to-volume ratio of 1:9 (g:mL). Subsequently, we centrifuged the homogenate at 2500 rpm for 10 min and then collected 0.1 mL of the supernatant. Following the manufacturer’s instructions from the Reduced Glutathione (GSH) Assay Kit (Njjcbio, Nanjing, China), we added 0.1 mL of the reagent and mixed it thoroughly. We then centrifuged the mixture at 3500 rpm for 10 min and collected the supernatant for colorimetric analysis.

### 3.10. Histology and Immunofluorescence Staining

Lung tissues were fixed with 4% paraformaldehyde for 48 h, routinely dehydrated, paraffin embedded, cut into 4 μm thick sections, stained with hematoxylin-eosin (H&E), and observed under light microscope for inflammatory cell infiltration in lung tissues. The expression of glutathione peroxidase 4 (GPX4) in the lung tissue was detected via an immunofluorescence assay using anti-GPX4 antibody. After washing, the slides were incubated with Alexa Fluor 488-conjugated goat anti-mouse (Lot: A10680) and CY3-conjugated goat anti-rabbit secondary antibody (Lot: A10520, Thermo Fisher Scientific). Slides were washed with PBS and counterstained with 4′,6-Diamidino-2-Phenylindole (DAPI). Fluorescence images were obtained using a whole-slide imaging system.

As described in our previous study [14]. Microbiota analysis was conducted using the Majorbio Cloud Platform (https://cloud.majorbio.com/; access date: 7 October 2024). α- and β-diversity, along with bacterial community composition, were assessed using the Quantitative Insights Into Microbial Ecology (QIIME) pipeline. Additionally, the linear discriminant analysis (LDA) effect size (LEfSe) was employed with a threshold LDA score >3.0 to identify differentially abundant taxa.

### 3.11. UPLC-MRM Analysis

The analysis of active components in serum samples was carried out using Waters Synapt G2-Si High-Definition Mass Spectrometry (HDMS) with H-class ultra-performance liquid chromatography (UPLC) and an Acquity Bridged Ethylene Hybrid (BEH) C18 column. Mobile phase A consisted of water containing 0.1% acetic acid, while mobile phase B comprised acetonitrile with 0.1% acetic acid. The solvent procedure is shown in Table 1. The injection volume was 2 μL, and the column temperature was 30 °C. MS was operated in negative mode under MRM. Data acquisition was performed using MassLynx 4.2.

PUFAs and LMs analyses were performed on a SCIEX 5500plus mass spectrometer equipped with an Electrospray Ionization (ESI) source (Framingham, MA, USA) and a Thermo Scientific Dionex ultimate 3000 HPLC (Waltham, MA, USA). The Waters ACQUITY UPLC BEH C18 (1.7 μm, 2.1 × 50 mm) column was used. Mobile phase A consisted of water with 0.1% acetic acid, whereas mobile phase B was a mixture of acetonitrile and isopropanol in a 9:1 volume ratio. The solvent procedure is shown in Table 2. The flow rate was set at 0.4 mL/min, with the column temperature maintained at 40 °C. An injection volume of 5 μL was used, and the MS was operated in negative mode under MRM. All data were acquired and processed using SCIEX Analyst software (version 1.7.1).

### 3.12. UPLC-MS Analysis

PCs analysis was performed on a SCIEX 5500plus mass spectrometer equipped with an ESI source (MA, USA) and a Thermo Scientific Dionex ultimate 3000 HPLC (MA, USA). The Waters ACQUITY UPLC C8 (1.7 μm, 2.1 × 100 mm) column was used. Mobile phase A consisted of a mixture of acetonitrile and water (3:2 *v*/*v*) containing 0.1% acetic acid and 10 mM ammonium acetate. Mobile phase B was a blend of acetonitrile and isopropanol (1:9 *v*/*v*) with 0.1% acetic acid and 10 mM ammonium acetate. The solvent procedure is shown in Table 3. The flow rate was set at 0.3 mL/min and the column temperature was maintained at 55 °C. The injection volume was 2 μL. MS was operated in positive mode under full scans. All data were acquired and processed using SCIEX Analyst software (version 1.7.1).

### 3.13. GC-MS Analysis

The SCFA analysis was conducted on an Agilent 7890B (Santa Clara, CA, USA) equipped with an automatic sampler (7693A) and coupled to an Agilent 5973 mass selective detector. The column employed was a fused-silica capillary column featuring a free fatty acid phase (DB-FFAP 122-3232) with dimensions of 0.25 mm internal diameter, 30 m length, and 0.25 μm film thickness. The helium carrier gas flow rate was set at 1 mL/min. The oven temperature was initially set at 50 °C and held for 1.5 min. It was then ramped up to 90 °C at a rate of 20 °C/min and held for 2.1 min. Subsequently, it was increased to 120 °C at a rate of 5 °C/min and held for 1 min. Finally, it was raised to 180 °C at a rate of 25 °C/min and held for an additional 1.5 min. The injected sample volume for the GC analysis was 0.5 μL, and the run time for each analysis was 16.5 min. The detector was operated in the selection ionization mode (SIM). Ion selection of the SCFAs was based on the retention time of standard compounds. All data were acquired and processed using Agilent MassHunter (version B.07.01).

### 3.14. Data Processing and Statistical Analysis

The open-source tool MetaboAnalyst 6.0 (available at http://www.metaboanalyst.ca/; access date: 15 January 2025) was utilized for the statistical analysis. The threshold for selecting important predictive variables was based on the variable importance in projection (VIP) score. Multivariate statistical differences were identified using an independent sample *t*-test. Variables with a VIP score greater than 1 and a *p*-value less than 0.05 were considered significant and selected as differential variables.

## 4. Discussion

In this study, ICGAC, identified as the key active ingredient in SXCF (a traditional folk remedy historically used for asthma relief in China), was assessed in an allergic asthma mouse model. Both dexamethasone and ICGAC significantly reduced typical lung morphological changes in asthma, such as pronounced inflammatory cell infiltration, bronchial epithelial degeneration, and interstitial inflammation. Remarkably, ICGAC exhibited therapeutic efficacy comparable to or exceeding that of dexamethasone, while demonstrating no experimentally detectable adverse effects commonly associated with steroid use. This highlights ICGAC’s potential as a safe and effective natural therapeutic option for managing allergic asthma.

### 4.1. Oral ICGAC Boosts Gut-Microbiota-Derived CGA and SCFAs as Bioactive Metabolites

We discovered that orally administered ICGAC is not the direct effective component; instead, it undergoes transformation into Chlorogenic acid. This transformation process is mediated by the gut microbiota, a complex community of microorganisms that play crucial physiological roles in food digestion, nutrient absorption, drug metabolism, and maintenance of gut barrier function. Upon entering the intestine, ICGAC may interact with these microorganisms, leading to its conversion into CGA.

Although the involvement of the gut microbiota in such transformation processes is well recognized, the specific bacterial species or strains responsible for converting ICGAC to CGA remain unclear. However, several studies have emphasized the significant role of the gut microbiota in the biotransformation of bioactive compounds [21]. For example, certain gut bacteria are capable of hydrolyzing, reducing, or methylating compounds, thereby altering their chemical structures and biological activities [22]. Our study detected a significant increase in the *Eubacterium_brachy_group*, suggesting its potential role in the conversion of ICGAC to CGA through analogous microbial metabolic pathways. To date, several studies have demonstrated that chlorogenic acid exhibits anti-inflammatory effects via the modulation of the intestinal microbiota [23].

Natural compounds frequently undergo structural modifications during absorption. Moreover, the effective form and pharmacological mechanisms of natural drugs can vary between single-dose and prolonged administrations [24]. Further research is essential to fully elucidate the intricate interactions between natural products, gut microbiota, and therapeutic efficacy.

Using UPLC-tandem mass spectrometry, we measured serum ICGAC and chlorogenic acid levels. Both H-ICGAC and L-ICGAC groups showed similar chlorogenic acid concentrations in the bloodstream (Figure 2A). This similarity can be attributed to the enhanced expression of P-glycoprotein (PGP) in the gut endothelial cells of the H-ICGAC group, facilitated by ICGAC.

PGP, an ATPase efflux pump that is a member of the ATP-binding cassette transporter family, is recognized for its ability to extrude a variety of lipophilic drugs. Contemporary studies have underscored a significant association between gut microbiota and PGP expression within the colon [25]. Specifically, *Clostridium* and *Bacillus* have been identified as key in stimulating PGP expression in the gut epithelium of mouse models, contributing to the observed correlation [25]. This suggests that ICGAC may upregulate PGP, leading to stable chlorogenic acid serum levels irrespective of the initial dose administered.

ICGAC also enhances the synthesis of SCFAs—notably, acetic acid and butyric acid—upon administration. SCFAs play a crucial role in modulating the immune system and reducing inflammation [26]. They also regulate various immune cells and influence T and B cell differentiation, impacting both innate and adaptive responses [27]. Furthermore, SCFAs reduce airway inflammation, a hallmark feature of allergic asthma [14].

Indeed, multiple studies emphasize the connection between SCFAs and allergic asthma. Changes in the gut microbiota, which produce SCFAs, can influence the risk of developing allergic asthma [14]. While, early-life antibiotic exposure disrupts the gut microbiota, increasing the risk of asthma and other allergic conditions [28]. Moreover, SCFAs modulate immune-related genes, potentially contributing to allergic asthma [29]. Intriguingly, our findings further show a positive correlation between butyrate production and PGP expression, highlighting its importance. A detailed analysis revealed that *Eubacterium_brachy_group* plays a key role in SCFA production following ICGAC administration (Figure 3).

### 4.2. ICGAC Modulates ALOX Activity and LMs to Reduce Asthmatic Inflammation

We explored the regulatory role of ICGAC in LMs metabolism and its anti-inflammatory potential through Lipidomics analysis. Our investigation focused on the activities of arachidonate lipoxygenases (ALOX-isoforms), particularly ALOX12 and ALOX15, which catalyze the oxidation of polyunsaturated fatty acids (PUFAs) and play crucial roles in inflammatory responses [30].We found that ICGAC treatment inhibited ALOX12 and ALOX15 activities, specifically reducing the enzyme-catalyzed oxidation of DHA (Figure 6A). DHA, an omega-3 PUFA, serves as a precursor for anti-inflammatory metabolites such as 11-HDoHE, 14-HDoHE, 16-HDoHE, 17-HDoHE, and RVD2, which are crucial in resolving inflammation in allergic asthma [31]. Disruption of their balance can result in heightened inflammatory reactions and asthma symptoms.

Consistently with this, chlorogenic acid, the active compound in ICGAC, has also been identified as a competitive inhibitor of ALOX, thereby inhibiting lipid peroxidation [32]. Taken together, our findings indicate that ICGAC modulates the metabolic networks of LMs and PUFAs, potentially exerting anti-inflammatory effects by regulating ALOX activity and LMs levels.

### 4.3. ICGAC Enhances GPX4, SLC7A11, and Nrf2 to Strengthen Antioxidant Defense

In this study, we explored how ICGAC suppresses asthma-related inflammation and oxidative stress, specifically by upregulating GPX4, SLC7A11, and Nrf2 in lung tissues.

Firstly, GPX4, an enzyme found on the outer surface of the inner mitochondrial membrane and within the mitochondrial matrix, is unique in its ability to directly reduce phospholipid peroxides (PL-OOH) in the mitochondrial membrane [33]. By using GSH as a co-substrate, GPX4 plays a vital role in regulating cellular lipid peroxides. Our findings revealed that the ICGAC treatment markedly increased GPX4 expression (Figure 6A), thereby enhancing the uptake and reduction in the peroxide load in PL-OOH, converting it into nontoxic lipid alcohol while oxidizing glutathione (reduced, GSH) to glutathione (oxidized, GSSG) [34].

Secondly, ICGAC significantly upregulated SLC7A11 expression (Figure 6A), which aids in the uptake of extracellular cystine for conversion to cysteine, a crucial precursor for glutathione synthesis [35]. Consistently with this upregulation, we observed a notable increase in GSH content in the lung tissue of ICGAC-treated mice compared to the model group.

Additionally, Nrf2, a member of the basic leucine zipper transcription factor family, is a central regulator of the antioxidant response. GPX4, a well-known transcriptional target of Nrf2, is upregulated by Nrf2 activation, creating a positive feedback loop that enhances cellular antioxidant defenses [36]. Our results indicated that ICGAC also increased Nrf2 and GPX4 expression (Figure 6A), further strengthening the antioxidant response.

**Figure 7:**
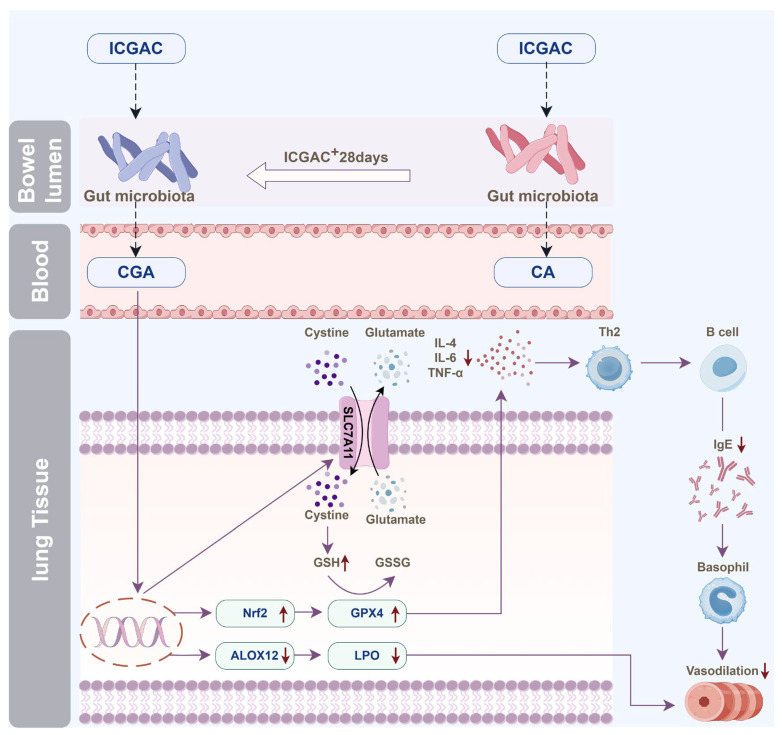
Illustration of the anti-asthmatic mechanism of ICGAC through the gut–lung axis. (The process of the change in the effective substances is related to the gut microbiota in mice. When ICGAC is administered for a long time, ICGAC in mice will be converted into CGA; when administered once, ICGAC will be converted into CA. ICGAC significantly upregulated the expression of SLC7A11 in mouse lung tissue. SLC7A11 helps to absorb cystine outside the cell and convert it into cysteine. ICGAC also upregulated the expression of GPX4 in mouse lung tissue. It reduced the release of inflammatory factor IL-4, thereby reducing the release of IgE by B cells. ICGAC also increased the expression of Nrf2, further strengthening the antioxidant response. CGA can inhibit the expression of ALOX12, thereby reducing the generation of inflammatory mediators such as 11-HDoHE, 14-HDoHE, 16-HDoHE, 17-HDoHE and RvD2, and alleviating asthma inflammation.).

Moreover, dendritic cells (DCs) facilitate the differentiation of naive CD4^+^ T cells to Th2 cells, which further stimulate B cells to produce IgE [37]. IL-4 amplifies Th2 activity, but elevated levels of Nrf2 inhibit its release [38]. ICGAC suppressed IL-4 and other asthma-related cytokines, thus reducing IgE production by B cells and alleviating asthma symptoms by lowering airway responsiveness (Figure 7).

## 5. Conclusions

In conclusion, our study integrates lipid metabolomics and network pharmacology to elucidate the mechanisms underlying ICGAC’s efficacy in treating allergic asthma. We demonstrate that, through gut microbiota-derived chlorogenic acid and SCFAs, ICGAC inhibits lipid peroxidation and inflammatory mediators. Our findings also highlight the critical roles of the GPX4 and Nrf2 pathways in ICGAC’s therapeutic effects. This research provides a robust foundation for the potential clinical application of ICGAC in asthma management and paves the way for future research and therapeutic innovations.

## Figures and Tables

**Table 1:** Gradient elution program for active components analysis.

Time (min)	A%	B%
0	95	5
1	95	5
4	40	60
4.5	0	100
5	0	100
5.1	95	5

**Table 2:** Gradient elution program for the PUFA and LM analysis.

Time (min)	A%	B%
0	75	25
1	75	25
8	5	95
10	5	95
10.01	75	25
12	75	25

**Table 3:** Gradient elution program for the PC analysis.

Time (min)	A%	B%
0	80	20
2	75	25
2.1	70	30
12	65	35
12.1	30	70
18	1	99
18.1	80	20
20	80	20

## Data Availability

Data is unavailable due to privacy or ethical restrictions.

## Supplementary Materials

The following supporting information can be downloaded at: https://www.mdpi.com/article/10.3390/ijms26104864/s1.

## List of Abbreviations

DEX, Dexamethasone; ICGAC, Isochlorogenic acid C; LMs, Lipid mediators; IgE, Immunoglobulin E; qRT-PCR, Quantitative reverse transcription PCR; OVA, Ovalbumins; TNF-α, Tumor necrosis factor-α; IL-1β, Interleukin-1β; IL-4, Interleukin-4; IL-5, Interleukin-5; IL-6, Interleukin-6; PCs, phosphatidyl cholines; PUFAs, polyunsaturated fatty acids; BALF, Bronchoalveolar lavage fluid; SCFAs, Bronchoalveolar lavage fluid; UHPLC, Ultra High Performance Liquid Chromatography; MRM, Multiple Reaction Monitoring; HRMS, High Resolution Mass Spectrometry; MTBE, methyl tert-butyl ether; BAS, Basophil; EOS, Eosinophil; WBC, White blood cell; LYM, Lymphocyte; MON, Monocytes; GSH, Glutathione; H&E, hematoxylin-eosin; GPX4, Glutathione Peroxidase 4; SPE, Solid-Phase Extraction; GC-MS, Gas Chromatography-Mass Spectrometry; BHT, Butylated hydroxytoluene; LDA, Linear Discriminant Analysis; LEfSe, LDA Effect Size; VIP, Variable Importance in Projection; Nrf2, Nuclear factor erythroid 2-related factor 2; ALOX12, Arachidonate 12-Lipoxygenase; DC, Dendritic cells; PCoA, Principal coordinate analysis; NMDS, Non-metric multidimensional scaling; ARA, Arachidonic acid; DHA, Docosahexaenoic Acid; DGLA, Dihomo-γ-linolenic acideicosapentaenoic Acid; EPA, Eicosapentaenoic Acid; LA, Linoleic Acid; PCA, Principal Component Analysis; RVD2, Resolvin D2; PLA2, Phospholipase A2 enzymes; SLC7A11, Solute Carrier Family 7 Member 11; SXCF, Hyssopus cuspidatus Boriss extract; ELASA, Enzyme linked immunosorbent assay; CGA, Chlorogenic acid; CA, Caffeic acid; PGP, P-glycoprotein.
