# Supplementary material for: Isochlorogenic Acid C Alleviates Allergic Asthma via Interactions Between Its Bioactive Form and the Gut Microbiome
Source: Int J Mol Sci. 2025 May 19;26(10):4864. doi: 10.3390/ijms26104864 (PMC12112207; doi:10.3390/ijms26104864)
Supplement: Supplementary file 1 [file ijms-26-04864-s001.zip › ijms-3565425-supplementary.pdf]

Isochlorogenic acid C alleviates allergic asthma via interaction between its bioactive form and the gut microbiome

Jing-Yi Xu [1#], Xiao-Juan Rong [2#], Zhen Shen [3], Yun-Dan Guo [3], Yi-Xuan Zhang [1], Chen-Chen Ding [1], Yi Wang [1], Yan-Xing Han [3], Tian-Le Gao [3*] and Cai Tie [1*]

1. State Key Laboratory for Fine Exploration and Intelligent Development of Coal Resources & School of Chemical and Environmental Engineering, China University of Mining and Technology-Beijing, Ding11 Xueyuan Road, Beijing 100083, China
2. Xinjiang Institute of Material Medica, Urumqi, Xinjiang 830004, China
3. State Key Laboratory of Bioactive Substances and Function of Natural Medicine, Institute of Materia Medica, Chinese Academy of Medical Sciences & Peking Union Medical College, Beijing 100050, China

#J.X. and X.R. contributed equally to this work.

* Correspondence: authors

* Correspondence: authors email: tianlegao@imm.ac.cn, & tiecai@cumtb.edu.cn

Analysis of pathological findings of lung injury in mice

Combined with the pathological histological changes of the lung in this test, the degree of lung injury was graded by using bronchial and perivascular inflammatory cell infiltration, bronchial obstruction, and interstitial inflammatory cell infiltration in the lung as the main observation indicators.

"-" Normal lung tissue morphology, no abnormal changes were observed.

"+" Minor (mild) pathological changes in a few parts of lung tissues (bronchi, interstitial lung).

"++" Moderate (moderate) pathological changes in some lung tissues (bronchi, interstitial lung).

"++++" Significant (severe) pathological changes in most lung tissues (bronchi, interstitial lung).

Table S1. Analysis of pathological findings of lung injury in mice

**Groups (n=6)**	**Classification of the degree of lung injury**			
	—	+	++	+++
CON (n=6)	5	1	0	0
MOD (n=8)	0	1	6	1
L-ICGAC (n=8)	0	1	7	0
H-ICGAC (n=8)	0	6	2	0
DEX (n=8)	0	5	3	0
